# RETRACTED: Mano, T. Application of Repetitive Transcranial Magnetic Stimulation over the Dorsolateral Prefrontal Cortex in Alzheimer’s Disease: A Pilot Study. *J. Clin. Med.* 2022, *11*, 798

**DOI:** 10.3390/jcm14031002

**Published:** 2025-02-05

**Authors:** Tomoo Mano

**Affiliations:** 1 Department of Rehabilitation Medicine, Nara Medical University, Nara 634-8521, Japan; manoneuro@naramed-u.ac.jp; 2 Department of Neuromodulation and Neurosurgery, Osaka University Graduate School of Medicine, Osaka 565-0871, Japan

The journal retracts the article titled “Application of Repetitive Transcranial Magnetic Stimulation over the Dorsolateral Prefrontal Cortex in Alzheimer’s Disease” [1], cited above.

Following publication, the author raised concerns to the attention of the publisher regarding an overlap between this article [1] and an earlier publication [2] published in another language.

Adhering to our standard procedure, an investigation was conducted by the Editorial Office and Editorial Board Member that confirmed the overlap between these two publications [1,2], and the lack of appropriate permission from the original publisher to republish. As a result, the Editorial Board and the author have decided to retract this publication [1], as per MDPI’s retraction policy (https://www.mdpi.com/ethics#_bookmark30, accessed on 30 September 2024).

This retraction was approved by the Editor-in-Chief of the *Journal of Clinical Medicine*.

The author agreed to this retraction.
